# (*E*)-*N*′-(4-Bromo­benzyl­idene)-3,4-dihydroxy­benzohydrazide monohydrate

**DOI:** 10.1107/S1600536809036964

**Published:** 2009-09-19

**Authors:** Dan-Yu Zhao, Chuan-Xun Li, Shan-shan Huang, Min-Tao Zhong, Hou-Li Zhang

**Affiliations:** a Liaoning University of Traditional Chinese Medicine, Shenyang, 110032, People’s Republic of China; b Dalian Medical University, Liaoning 116044, People’s Republic of China

## Abstract

In the title compound, C_14_H_11_BrN_2_O_3_·H_2_O, the dihedral angle between the two benzene rings of the Schiff base is 22.7 (2)° and an intra­molecular O—H⋯O hydrogen bond is observed. In the crystal, mol­ecules are linked into layers parallel to the *ab* plane by O—H⋯O and N—H⋯O hydrogen bonds.

## Related literature

For the synthesis of Schiff base compounds from the reaction of aldehydes with primary amines, see: Herrick *et al.* (2008 ▶); Suresh *et al.* (2007 ▶). For a related structure, see: Ma *et al.* (2008 ▶). For reference structural data, see: Allen *et al.* (1987 ▶).
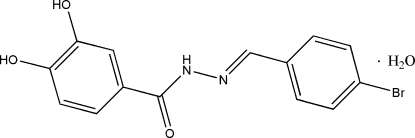


## Experimental

### 

#### Crystal data

C_14_H_11_BrN_2_O_3_·H_2_O
*M* *_r_* = 353.17
Monoclinic, 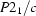

*a* = 7.8119 (5) Å
*b* = 13.8504 (9) Å
*c* = 13.0764 (9) Å
β = 91.708 (1)°
*V* = 1414.21 (16) Å^3^
*Z* = 4
Mo *K*α radiation
μ = 2.92 mm^−1^
*T* = 295 K
0.18 × 0.16 × 0.15 mm

#### Data collection

Siemens SMART CCD diffractometer
Absorption correction: multi-scan *SADABS* (Sheldrick, 1996 ▶) *T*_min_ = 0.621, *T*_max_ = 0.668
7384 measured reflections
2511 independent reflections
1810 reflections with *I* > 2σ(*I*)
*R*_int_ = 0.096

#### Refinement

*R*[*F* ^2^ > 2σ(*F* ^2^)] = 0.044
*wR*(*F* ^2^) = 0.116
*S* = 1.02
2511 reflections
192 parameters
H-atom parameters constrained
Δρ_max_ = 0.76 e Å^−3^
Δρ_min_ = −0.69 e Å^−3^

### 

Data collection: *SMART* (Siemens, 1996 ▶); cell refinement: *SAINT* (Siemens, 1996 ▶); data reduction: *SAINT*; program(s) used to solve structure: *SHELXS97* (Sheldrick, 2008 ▶); program(s) used to refine structure: *SHELXL97* (Sheldrick, 2008 ▶); molecular graphics: *SHELXTL* (Sheldrick, 2008 ▶); software used to prepare material for publication: *SHELXTL*.

## Supplementary Material

Crystal structure: contains datablocks global, I. DOI: 10.1107/S1600536809036964/hb5092sup1.cif

Structure factors: contains datablocks I. DOI: 10.1107/S1600536809036964/hb5092Isup2.hkl

Additional supplementary materials:  crystallographic information; 3D view; checkCIF report

## Figures and Tables

**Table 1:** Hydrogen-bond geometry (Å, °)

*D*—H⋯*A*	*D*—H	H⋯*A*	*D*⋯*A*	*D*—H⋯*A*
O1—H1⋯O2	0.82	2.30	2.734 (3)	114
O4—H16⋯O2^i^	0.85	2.03	2.760 (3)	143
O4—H15⋯O3^ii^	0.85	1.94	2.761 (3)	163
O2—H2⋯O3^iii^	0.82	1.91	2.675 (3)	154
O1—H1⋯O4^iv^	0.82	2.16	2.929 (4)	155
N1—H1*A*⋯O4^v^	0.86	2.07	2.898 (4)	162

Symmetry codes: (i) 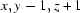; (ii) 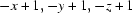; (iii) 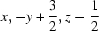; (iv) 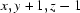; (v) 
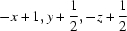
.

**Fig. 1.**
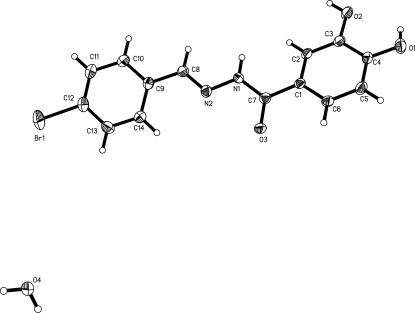
The molecular structure of (I), with displacement ellipsoids drawn at the 30% probability level. The dashed lines indicate hydrogen bonds.

**Fig. 2.**
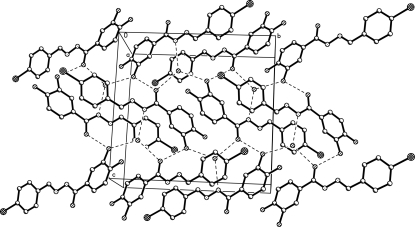
The molecular packing of (I). Intermolecular hydrogen bonds are shown as dashed lines. H atoms not involved in the hydrogen bonds have been omitted for clarity.

## supplementary crystallographic information

### Comment

Schiff base compounds can be easily synthesized from the reaction of aldehydes with primary amines (Herrick *et al.*, 2008; Suresh *et al.*, 2007). In this paper, the crystal structure of a new Schiff base compound derived from the condensation reaction of 3,4-dihydroxybenzohydrazide with 4-bromobenzaldehyde is reported.

The Schiff base molecule of the title compound, (I), displays a *trans* configuration with respect to the C=N and C—N bonds (Fig. 1). All the bond lengths are within normal ranges (Allen *et al.*, 1987), and are comparable to those in the related compound 3,4-Dihydroxy-*N'*-(2-hydroxybenzylidene)benzohydrazide- methanol-water (2/1/3) (Ma *et al.*, 2008). The dihedral angle between the two benzene rings in (I) is 22.7 (2)°. An intramolecular O—H···O hydrogen bond is observed. In the crystal structure the water molecule links three symmetry related molecules through O—H···O and O—H···N hydrogen bonds (Table 1). Together with two further intermolecular O—H···O hydrogen bonds, layers parallel to the *ab* plane are formed (Fig. 2).

### Experimental

4-Bromosalicylaldehyde (0.1 mmol, 15.6 mg) and 3,4-dihydroxybenzoic acid hydrazide (0.1 mmol, 16.8 mg) were dissolved in a 95% ethanol solution (10 ml). The mixture was stirred at room temperature to give a clear colorless solution. Light yellow blokcs of (I) were formed by gradual evaporation of the solvent over a period of nine days at room temperature.

### Refinement

All H atoms were placed in geometrically idealized positions (C—H = 0.93 Å, O—H = 0.82–0.85 Å and N—H = 0.86 Å) and refned as riding with *U*_iso_(H) = 1.2*U*_eq_(C,*N*) or 1.5*U*_eq_(O).

### Crystal data

**Table:**

C_14_H_11_BrN_2_O_3_·H_2_O	*F*(000) = 712
*M**_r_* = 353.17	*D*_x_ = 1.659 Mg m^−^^3^
Monoclinic, *P*2_1_/*c*	Mo *K*α radiation, λ = 0.71073 Å
Hall symbol: -P 2ybc	Cell parameters from 2538 reflections
*a* = 7.8119 (5) Å	θ = 2.7–24.2°
*b* = 13.8504 (9) Å	µ = 2.92 mm^−^^1^
*c* = 13.0764 (9) Å	*T* = 295 K
β = 91.708 (1)°	Block, light yellow
*V* = 1414.21 (16) Å^3^	0.18 × 0.16 × 0.15 mm
*Z* = 4	

### Data collection

**Table:**

Siemens SMART CCD diffractometer	2511 independent reflections
Radiation source: fine-focus sealed tube	1810 reflections with *I* > 2σ(*I*)
graphite	*R*_int_ = 0.096
φ and ω scans	θ_max_ = 25.0°, θ_min_ = 2.1°
Absorption correction: multi-scan *SADABS* (Sheldrick, 1996)	*h* = −9→9
*T*_min_ = 0.621, *T*_max_ = 0.668	*k* = −12→16
7384 measured reflections	*l* = −12→15

### Refinement

**Table:**

Refinement on *F*^2^	Primary atom site location: structure-invariant direct methods
Least-squares matrix: full	Secondary atom site location: difference Fourier map
*R*[*F*^2^ > 2σ(*F*^2^)] = 0.044	Hydrogen site location: inferred from neighbouring sites
*wR*(*F*^2^) = 0.116	H-atom parameters constrained
*S* = 1.02	*w* = 1/[σ^2^(*F*_o_^2^) + (0.053*P*)^2^ + 0.0453*P*] where *P* = (*F*_o_^2^ + 2*F*_c_^2^)/3
2511 reflections	(Δ/σ)_max_ < 0.001
192 parameters	Δρ_max_ = 0.76 e Å^−^^3^
0 restraints	Δρ_min_ = −0.69 e Å^−^^3^

### Special details

**Table:**

Geometry. All e.s.d.'s (except the e.s.d. in the dihedral angle between two l.s. planes) are estimated using the full covariance matrix. The cell e.s.d.'s are taken into account individually in the estimation of e.s.d.'s in distances, angles and torsion angles; correlations between e.s.d.'s in cell parameters are only used when they are defined by crystal symmetry. An approximate (isotropic) treatment of cell e.s.d.'s is used for estimating e.s.d.'s involving l.s. planes.
Refinement. Refinement of *F*^2^ against ALL reflections. The weighted *R*-factor *wR* and goodness of fit *S* are based on *F*^2^, conventional *R*-factors *R* are based on *F*, with *F* set to zero for negative *F*^2^. The threshold expression of *F*^2^ > σ(*F*^2^) is used only for calculating *R*-factors(gt) *etc*. and is not relevant to the choice of reflections for refinement. *R*-factors based on *F*^2^ are statistically about twice as large as those based on *F*, and *R*- factors based on ALL data will be even larger.

### Fractional atomic coordinates and isotropic or equivalent isotropic displacement parameters (Å^2^)

**Table:**

	*x*	*y*	*z*	*U*_iso_*/*U*_eq_	
Br1	1.08523 (6)	0.16874 (3)	0.20512 (4)	0.0710 (2)	
O1	0.2102 (4)	1.04968 (18)	−0.1288 (2)	0.0561 (7)	
H1	0.2496	1.0583	−0.1855	0.084*	
O2	0.4375 (4)	0.94026 (17)	−0.23369 (19)	0.0590 (8)	
H2	0.4882	0.8962	−0.2617	0.089*	
O3	0.4997 (3)	0.72271 (16)	0.16417 (17)	0.0439 (6)	
O4	0.4061 (3)	0.12989 (17)	0.70360 (19)	0.0500 (7)	
H15	0.4361	0.1660	0.7536	0.075*	
H16	0.4622	0.0787	0.7172	0.075*	
N1	0.5896 (3)	0.66104 (17)	0.0155 (2)	0.0349 (6)	
H1A	0.5932	0.6652	−0.0500	0.042*	
N2	0.6628 (3)	0.58384 (19)	0.0659 (2)	0.0364 (7)	
C1	0.4411 (4)	0.8144 (2)	0.0139 (2)	0.0310 (7)	
C2	0.4779 (4)	0.8365 (2)	−0.0866 (3)	0.0343 (8)	
H2A	0.5545	0.7982	−0.1215	0.041*	
C3	0.4030 (5)	0.9141 (2)	−0.1350 (3)	0.0379 (8)	
C4	0.2885 (4)	0.9734 (2)	−0.0836 (3)	0.0375 (8)	
C5	0.2535 (4)	0.9517 (2)	0.0159 (3)	0.0435 (9)	
H5	0.1770	0.9901	0.0508	0.052*	
C6	0.3290 (4)	0.8743 (2)	0.0652 (3)	0.0378 (8)	
H6	0.3050	0.8619	0.1331	0.045*	
C7	0.5118 (4)	0.7306 (2)	0.0699 (3)	0.0335 (7)	
C8	0.7358 (4)	0.5211 (2)	0.0103 (3)	0.0357 (8)	
H8	0.7339	0.5285	−0.0604	0.043*	
C9	0.8220 (4)	0.4381 (2)	0.0570 (3)	0.0344 (8)	
C10	0.8858 (4)	0.3655 (2)	−0.0043 (3)	0.0419 (9)	
H10	0.8742	0.3708	−0.0750	0.050*	
C11	0.9666 (4)	0.2852 (2)	0.0382 (3)	0.0456 (9)	
H11	1.0085	0.2367	−0.0034	0.055*	
C12	0.9835 (4)	0.2786 (2)	0.1423 (3)	0.0423 (9)	
C13	0.9267 (5)	0.3516 (3)	0.2044 (3)	0.0454 (9)	
H13	0.9435	0.3474	0.2750	0.054*	
C14	0.8455 (4)	0.4301 (2)	0.1623 (3)	0.0412 (8)	
H14	0.8056	0.4786	0.2045	0.049*	

### Atomic displacement parameters (Å^2^)

**Table:**

	*U*^11^	*U*^22^	*U*^33^	*U*^12^	*U*^13^	*U*^23^
Br1	0.0630 (3)	0.0516 (3)	0.0988 (5)	0.02020 (19)	0.0116 (3)	0.0259 (2)
O1	0.0711 (18)	0.0459 (15)	0.0516 (17)	0.0227 (13)	0.0093 (14)	0.0098 (13)
O2	0.114 (2)	0.0323 (13)	0.0316 (14)	0.0185 (14)	0.0206 (14)	0.0067 (11)
O3	0.0707 (16)	0.0354 (13)	0.0260 (14)	0.0040 (11)	0.0064 (12)	−0.0001 (10)
O4	0.0816 (18)	0.0311 (12)	0.0372 (14)	0.0045 (12)	−0.0024 (13)	0.0006 (11)
N1	0.0483 (16)	0.0296 (14)	0.0268 (15)	0.0035 (12)	0.0031 (12)	0.0025 (12)
N2	0.0420 (16)	0.0286 (14)	0.0386 (17)	0.0000 (12)	0.0029 (13)	0.0034 (13)
C1	0.0393 (17)	0.0260 (15)	0.0277 (18)	−0.0034 (13)	0.0021 (14)	−0.0021 (14)
C2	0.0491 (19)	0.0243 (16)	0.0302 (19)	0.0000 (14)	0.0104 (15)	−0.0018 (14)
C3	0.058 (2)	0.0268 (17)	0.0290 (19)	−0.0029 (15)	0.0086 (16)	0.0006 (15)
C4	0.0461 (19)	0.0287 (17)	0.038 (2)	0.0047 (15)	0.0014 (16)	−0.0007 (15)
C5	0.050 (2)	0.040 (2)	0.042 (2)	0.0093 (16)	0.0136 (17)	−0.0038 (17)
C6	0.048 (2)	0.0382 (18)	0.0273 (18)	0.0036 (16)	0.0103 (15)	0.0009 (15)
C7	0.0406 (18)	0.0287 (17)	0.031 (2)	−0.0053 (14)	0.0025 (15)	−0.0014 (15)
C8	0.0424 (18)	0.0322 (18)	0.0326 (19)	−0.0023 (15)	0.0033 (15)	0.0000 (15)
C9	0.0321 (16)	0.0298 (17)	0.042 (2)	−0.0036 (13)	0.0041 (15)	0.0006 (15)
C10	0.046 (2)	0.0390 (19)	0.041 (2)	−0.0008 (16)	0.0039 (17)	−0.0066 (17)
C11	0.0411 (19)	0.0340 (19)	0.062 (3)	0.0026 (15)	0.0049 (18)	−0.0069 (18)
C12	0.0335 (18)	0.0343 (19)	0.059 (3)	0.0005 (14)	0.0078 (17)	0.0066 (18)
C13	0.047 (2)	0.052 (2)	0.037 (2)	0.0060 (17)	0.0031 (17)	0.0067 (18)
C14	0.046 (2)	0.0391 (19)	0.039 (2)	0.0044 (16)	0.0041 (16)	−0.0044 (16)

### Geometric parameters (Å, °)

**Table:**

Br1—C12	1.893 (3)	C3—C4	1.402 (5)
O1—C4	1.348 (4)	C4—C5	1.372 (5)
O1—H1	0.8200	C5—C6	1.374 (5)
O2—C3	1.374 (4)	C5—H5	0.9300
O2—H2	0.8200	C6—H6	0.9300
O3—C7	1.244 (4)	C8—C9	1.458 (5)
O4—H15	0.8499	C8—H8	0.9300
O4—H16	0.8500	C9—C10	1.387 (5)
N1—C7	1.353 (4)	C9—C14	1.387 (5)
N1—N2	1.372 (4)	C10—C11	1.387 (5)
N1—H1A	0.8600	C10—H10	0.9300
N2—C8	1.277 (4)	C11—C12	1.367 (5)
C1—C2	1.387 (5)	C11—H11	0.9300
C1—C6	1.393 (4)	C12—C13	1.378 (5)
C1—C7	1.471 (4)	C13—C14	1.367 (5)
C2—C3	1.369 (5)	C13—H13	0.9300
C2—H2A	0.9300	C14—H14	0.9300
			
C4—O1—H1	109.5	O3—C7—N1	120.5 (3)
C3—O2—H2	109.5	O3—C7—C1	121.6 (3)
H15—O4—H16	101.6	N1—C7—C1	117.9 (3)
C7—N1—N2	119.3 (3)	N2—C8—C9	120.4 (3)
C7—N1—H1A	120.3	N2—C8—H8	119.8
N2—N1—H1A	120.3	C9—C8—H8	119.8
C8—N2—N1	116.3 (3)	C10—C9—C14	118.4 (3)
C2—C1—C6	118.4 (3)	C10—C9—C8	119.9 (3)
C2—C1—C7	124.1 (3)	C14—C9—C8	121.6 (3)
C6—C1—C7	117.5 (3)	C11—C10—C9	121.1 (3)
C3—C2—C1	120.9 (3)	C11—C10—H10	119.4
C3—C2—H2A	119.5	C9—C10—H10	119.4
C1—C2—H2A	119.5	C12—C11—C10	118.8 (3)
C2—C3—O2	123.3 (3)	C12—C11—H11	120.6
C2—C3—C4	120.6 (3)	C10—C11—H11	120.6
O2—C3—C4	116.1 (3)	C11—C12—C13	121.0 (3)
O1—C4—C5	119.2 (3)	C11—C12—Br1	120.8 (3)
O1—C4—C3	122.5 (3)	C13—C12—Br1	118.2 (3)
C5—C4—C3	118.3 (3)	C14—C13—C12	119.9 (3)
C4—C5—C6	121.4 (3)	C14—C13—H13	120.0
C4—C5—H5	119.3	C12—C13—H13	120.0
C6—C5—H5	119.3	C13—C14—C9	120.7 (3)
C5—C6—C1	120.4 (3)	C13—C14—H14	119.7
C5—C6—H6	119.8	C9—C14—H14	119.7
C1—C6—H6	119.8		
			
C7—N1—N2—C8	−179.3 (3)	C6—C1—C7—O3	−13.4 (5)
C6—C1—C2—C3	−1.4 (5)	C2—C1—C7—N1	−13.4 (5)
C7—C1—C2—C3	177.9 (3)	C6—C1—C7—N1	165.9 (3)
C1—C2—C3—O2	178.7 (3)	N1—N2—C8—C9	178.0 (3)
C1—C2—C3—C4	0.6 (5)	N2—C8—C9—C10	173.5 (3)
C2—C3—C4—O1	−179.1 (3)	N2—C8—C9—C14	−7.8 (5)
O2—C3—C4—O1	2.7 (5)	C14—C9—C10—C11	2.0 (5)
C2—C3—C4—C5	−0.2 (5)	C8—C9—C10—C11	−179.3 (3)
O2—C3—C4—C5	−178.4 (3)	C9—C10—C11—C12	−0.3 (5)
O1—C4—C5—C6	179.5 (3)	C10—C11—C12—C13	−2.1 (5)
C3—C4—C5—C6	0.5 (5)	C10—C11—C12—Br1	177.7 (2)
C4—C5—C6—C1	−1.4 (5)	C11—C12—C13—C14	2.8 (5)
C2—C1—C6—C5	1.8 (5)	Br1—C12—C13—C14	−177.0 (3)
C7—C1—C6—C5	−177.5 (3)	C12—C13—C14—C9	−1.1 (5)
N2—N1—C7—O3	−3.3 (5)	C10—C9—C14—C13	−1.3 (5)
N2—N1—C7—C1	177.5 (2)	C8—C9—C14—C13	−180.0 (3)
C2—C1—C7—O3	167.3 (3)		

### Hydrogen-bond geometry (Å, °)

**Table:**

*D*—H···*A*	*D*—H	H···*A*	*D*···*A*	*D*—H···*A*
O1—H1···O2	0.82	2.30	2.734 (3)	114
O4—H16···O2^i^	0.85	2.03	2.760 (3)	143
O4—H15···O3^ii^	0.85	1.94	2.761 (3)	163
O2—H2···O3^iii^	0.82	1.91	2.675 (3)	154
O1—H1···O4^iv^	0.82	2.16	2.929 (4)	155
N1—H1A···O4^v^	0.86	2.07	2.898 (4)	162

Symmetry codes: (i) *x*, *y*−1, *z*+1; (ii) −*x*+1, −*y*+1, −*z*+1; (iii) *x*, −*y*+3/2, *z*−1/2; (iv) *x*, *y*+1, *z*−1; (v) −*x*+1, *y*+1/2, −*z*+1/2.
